# Wild Foods: A Topic for Food Pre-History and History or a Crucial Component of Future Sustainable and Just Food Systems?

**DOI:** 10.3390/foods10040827

**Published:** 2021-04-10

**Authors:** Andrea Pieroni

**Affiliations:** 1 University of Gastronomic Sciences, Piazza Vittorio Emanuele II 9, I-12042 Pollenzo/Bra (Cuneo), Italy; a.pieroni@unisg.it; 2 Department of Medical Analysis, Faculty of Science, Tishk International University, 44001 Erbil, Kurdistan, Iraq

The ethnobiology of wild foods has garnered increasing attention in food studies in recent years, since traditional foodways in less urbanized and globalized areas of the world are sometimes still based on often neglected or even largely unknown wild plant, animal, fungal, microorganism, and mineral ingredients, as well as their food products and culinary preparations. 

In recent decades, wild foods in different parts of the world have been the subject of historical and cultural heritage studies, as well as—increasingly for what specifically concerns wild food plants—evaluation studies in terms of their nutritional and nutraceutical assessment. Within these processes, wild foods are attracting immense attention from food activists and consumers, as well as chefs and gastronomic entrepreneurs, not to mention stakeholders working on food security, food sovereignty-centered policies, and sustainable diets and agro-ecology. This is especially due to the explosion of interest in foraging practices and to the attention paid by the entire gastronomic sector, which is bridging innovation and the (re)invention of wild food “traditions”. The current Special Issue, which is aimed at presenting original research on the biocultural aspects of wild foods, as well as their temporal and spatial dynamics, offers a broad spectrum of approaches and inspirational scientific investigations concerning this topic from diverse areas of the globe.

Of the five European case studies presented in this Special Issue, one investigates the Catalonian portion of the Mediterranean region, while four contributions focus on the Eastern fringe of the continent, and in particular, the Western border of the former Soviet Union. Gras et al. [1] provide an impressive and comprehensive review of Catalan wild plant food ingredients and identify 291 plants representing the “foraging” plant heritage, which the authors still consider largely underestimated and underutilized. The contribution also proposes that some of these “forgotten foods” could be newly introduced into the market, first, but not only, at a local level, which could be interesting for new crop development in the framework of further valorization of territorial identity.

On the Eastern European periphery, Kalle et al. [2] focus on the historical and contemporary trajectory of wild food plant gathering and processing among the Seto minority group in SE Estonia and analyze the possible effects of the state border established between Estonia and Russia in the early 1990s. The authors find that while the exchange of food knowledge and skills, as well as commercial activities around wild food plants, have been fading in recent decades, the popularity of a healthy lifestyle, however, seems to have introduced new wild food plant preparations.

Belichenko et al. [3] further analyze the wild plant ingredients used on the two sides of the Estonian–Russian border, and while finding substantial homogeneity for the most commonly used plants, the investigated communities appear ethnobotanically closer to the dominant ethnic groups immediately surrounding them than to those across the border.

Kolosova et al. [4] investigate wild food plant gathering in Karelia, NW Russia and observe that while throughout the lifetime of the interviewees the list of used wild items did not change considerably, the ways in which they are processed and stored underwent several stages as a function of centrally available goods, people’s welfare, technical progress, and ideas about the harm and benefit of various products and technological processes.

Stryamets et al.’s work focuses instead on the southern portion of the former western Soviet Union and investigates the contemporary use of wild food plants among Romanians divided for several decades by the border between Ukraine (formerly the Soviet Union) and Romania; the authors observe how wild food plant-centered jams, *sarma*, and homemade alcoholic beverages predominate among Romanians living in Romania, while tea, soups, and birch sap-based beverages are more common among Romanians living on the northern, Slavic side of the border [5]. The authors suggest that cross-ethnic marriages promoted by different state authorities during the past decades, as well as markets and women’s networks, i.e., “neighbors do so”, may have had a great impact on changes in wild food uses.

In Asia, the four case studies presented in this Special Issue touch on the south and SE portions of the continent. Aziz et al. [6] compare the use of wild plant ingredients among different linguistic and religious groups in a remote mountain area of Gilgit Baltistan in northern Pakistan. The authors observe that a rich food heritage in the use of wild plant ingredients still survives, but at the same time, this seems to be threatened by changes in lifestyle and globalization processes. Moreover, the documented wild food plant heritage is largely shared by the very diverse considered communities; the authors suggest that this may possibly be attributed to the cultural pluralism of the study area, fostered by the Ismaili branch of Islam, which may have enhanced cultural exchanges for centuries. Furthermore, the authors postulate that the recorded biocultural wild food heritage could represent a crucial driver, if properly revitalized, for assuring the food security of the local communities and also for further developing ecotourism and the associated sustainable gastronomic initiatives.

Majeed et al. [7] conducted extensive fieldwork on the eastern side of northern Pakistan in the Jhelum District of Punjab and compared the wild plants foraged by six religious groups; they found a high homogeneity of use among the two Muslim (Shia and Sunni) clusters, while the other four religious groups showed less extensive, yet diverse uses. Moreover, the field study reveals a remarkable erosion of the wild plant knowledge among the non-Muslim groups, which are more engaged in urban occupations and have possibly undergone stronger cultural adaption to a modern lifestyle. 

Jugli et al. [8] address the cultural value and ritual uses of wild food animals among the Tangsa and Wancho communities of Eastern Arunachal Pradesh (India). Very diverse uses of animals and their parts are used during rituals and festivals and their significance in decorations and adornments, in supernatural beliefs, and in connection with tribal folklore (stories) reveal the reasons some species are hunted and consumed while others, for no apparent reason, are killed or simply ignored. Furthermore, the authors suggest the crucial roles that the government as well as tribal leaders and the communities may play in managing animal biodiversity and in slowing the erosion and gradual disappearance of traditional knowledge.

In the southern portion of the Asian continent, Pawera et al. [9] document the diversity of wild plant ingredients and perceptions, attitudes, and drivers of change in their consumption among Minangkabau and Mandailing women farmers in West Sumatra, Indonesia. Respondents attributed the erosion of traditional skills in recognizing and processing wild plant foods to the decreased availability of wild plants and changes in their lifestyle (time constraints, and a limited knowledge of the nutritional value of wild plants); while the key motivations for their use were that they are perceived as “unpolluted natural foods”. The authors reflect that, despite socio–economic factors and changes in agriculture and markets, the persistence of a strong cultural link to wild plant foods appears to slow potential unhealthy dietary changes and as a result, communities, governments, and NGOs should work together to optimize their use in a sustainable way. 

Finally, Shai et al. [10] conducted an ethnobotanical survey on the wild fruits gathered and consumed in three villages of Mpumalanga Province, South Africa, and analyzed how these locally sourced ingredients still play a significant role in the daily lives of the Mapulana people. The authors suggested that the identified fruit species have the potential to be an important alternative food source to help meet the dietary requirements and health needs of communities, especially those in remote rural areas.

To summarize, the main findings of this Special Issue focus on the following four core concepts:

- Traditional knowledge and practices linked to wild foods are rapidly changing and are nowadays severely endangered by globalization processes, even in the most remote areas of the globe;
- Wild foods represent a complex and fascinating part of local bio–cultural and food heritage, with projections in terms of identity, values, and ecological and cultural meanings which go well beyond the food domain;
- A remarkable resurgence of interest in the procurement, processing, and consumption of wild foods is to be observed in some contexts, mainly promoted by the interest communities have in “natural”, local, and healthy foods;
- A substantial need for more systematic strategies of the bio–nutritional–pharmacological evaluation of these ingredients, as well as of their socio–economic valorization processes, is addressed by the scientific community.

Even though ethnography-centered field studies aimed at documenting wild foods in many remote areas of the world are still essential, the authors in this Special Issue suggest that there is an emerging need to (further) explore the ethnobiology of wild foods along the following trajectories:

- Cross-cultural comparisons focusing on different ethnic/linguistic/religious groups, in order to foster both ecological and social sustainability, so as to valorize those portions of the biocultural food heritage that are environmentally practicable in a time of global warming, as well as those specifically held by marginalized groups; and to frame all this within rural development policies devoted to shaping germane perspectives for threatened peripheral communities and environments;
- Temporal comparisons employing both ethnographic and historical analysis, with particular attention paid to the issue of the mobility of goods and people (e.g., “newcomers” and migrant communities), in order to provide more inspiring instruments for realizing peaceful social co-existence;
- Social media and its role in new “alphabetization processes” in foraging, hunting, and fishing, i.e., the contemporary foraging-euphoria and new sustainable gastronomies;
- Sensory and cognitive analysis on cultural perceptions and preferences of wild foods, in order to see how “old” and new consumer behaviors can contribute to the valorization of wild foods;
- Studies on the socio–economic valorization processes of wild foods, i.e., investigating the ambiguous nature of their commodification that can sometimes lead, in different contexts, to over-harvesting and may in some cases even threaten the continuation of their use.

Subsequent years and decades will certainly tell us more about the future of wild foods in scientific research and in societies as well, and how these old historical food trajectories will continue to leave their irreplaceable and also fascinating traces in our dishes, food environments, cultures, and aesthetics.

## Data Availability

Not applicable.
